# UV-Induced Photodegradation of Naproxen Using a Nano γ-FeOOH Composite: Degradation Kinetics and Photocatalytic Mechanism

**DOI:** 10.3389/fchem.2019.00847

**Published:** 2019-12-12

**Authors:** Zhanyi Li, Guoguang Liu, Qing Su, Chunyan Lv, Xiaoyu Jin, Xiaoqing Wen

**Affiliations:** ^1^School of Environmental Science and Engineering, Guangdong University of Technology, Guangzhou, China; ^2^School of Computer Science and Technology, Guangdong University of Technology, Guangzhou, China; ^3^Department of Materials Chemistry, Huzhou University, Huzhou, China

**Keywords:** γ-FeOOH, NPX, photodegradation, photocatalysis, active substances

## Abstract

Naproxen (NPX) is one of the most common pharmaceutical and personal care products found in surface water, which is recalcitrant to degradation by biological treatment or complete removal via traditional sewage treatment processes. In this study, nanoscale γ-FeOOH was synthesized and characterized by X-ray diffraction, scanning electron microscopy, surface analysis, and analysis of the forbidden bandwidth. Under UV irradiation, γ-FeOOH had the capacity to rapidly photodegrade NPX. The photodegradation rate of NPX was dependent on the concentration of γ-FeOOH in solution, initial NPX concentration, and pH. By increasing the concentration of γ-FeOOH, the NPX photodegradation rate was increased and then remained stable. Furthermore, the highest photodegradation rate for NPX was observed under acidic conditions. Through the analysis of the active substances (such as h^+^, e^−^, OH, ^1^O_2_, and ${\text{O}}_{2}^{\cdot -}$) by electron spin resonance, the photocatalytic mechanism of NPX degradation on γ-FeOOH was determined to be semiconductor photocatalysis.

## Introduction

With continuous improvements in anthropogenic living standards, the contamination of natural waterways has become an unavoidable and often neglected environmental issue. At present, however, water monitoring standards do not include pharmaceutical and personal care products (PPCPs), which are recalcitrant to biodegradation or complete removal via traditional sewage treatment processing technologies (Christina et al., 2014). Concurrently, PPCPs are constantly released into the environment from the medical and livestock industries; hence, they have garnered the attention of researchers and the public due to their “pseudo persistence.” Common PPCPs include non-steroidal anti-inflammatory and analgesic drugs such as Ibuprofen, Naproxen, Aspirin, and Diclofenac. Naproxen (NPX) is a commonly used anti-inflammatory and analgesic with negligible side effects; thus, it is one of the four most commonly consumed prescriptions on a global scale (Jones et al., 2002). Although the NPX concentration in water is low, it has accumulated to ng/L concentration levels (Dai et al., 2014). Medical studies have revealed that the long-term ingestion of trace NPX levels can induce heart disease, stroke, and toxic pulmonary effects (Isidori et al., 2005; Karl et al., 2006; Domínguez et al., 2011; Hasan et al., 2012). Current NPX treatment methods encompass adsorption (Xu et al., 2009; Hasan et al., 2012) photocatalytic degradation (Méndez-Arriaga et al., 2008), and radiation (Zheng et al., 2011), which commonly employ FeOOH (the primary component of rust, a corrosion product on metal surfaces) (Molgaard, 1974). It has been reported that FeOOH exists not only in marine shellfish (Lee et al., 2000), but also within soils, sediments, and water, and the physical and chemical properties of FeOOH are stable. This compound possesses a relatively large surface area, which likely plays a critical role in the removal of contaminants from the natural environment (Fortin and Langley, 2005; Zhou et al., 2007); hence, it is receiving increased attention in the areas of environmental restoration and governance.

Nano-γ-FeOOH exhibits surface resident and interfacial effects as well as unique properties at the nanoscale and quantum levels (Nurmi et al., 2005). It can effectively adsorb organic matter in water and demonstrates a good flocculation effect. Under certain conditions of light and oxygen exposure, it may also catalyze the degradation of adsorbed organic matter without producing secondary pollution. At present, γ-FeOOH is mainly used for industrial desulfurization; thus, there have been few studies on the adsorption and photocatalysis of PPCP contaminants. Further, investigations of NPX-related adsorption and photocatalytic processes and mechanisms have rarely been reported.

For this investigation, nano-γ-FeOOH is synthesized, and on the basis of previous studies that examined its adsorption performance for NPX (Zhanyi et al., 2018), the primary focus here is centered on the effects of nano-γ-FeOOH on the photocatalytic degradation of NPX. The effects of the photocatalyst dosage, initial NPX concentration, pH, and other factors are investigated. This work culminates in the proposal of an environmentally compatible photocatalytic strategy for the effective treatment of NPX-infused wastewater.

## Experiment

### Materials and Reagents

NPX (A-methyl-6-methoxy-2-naphthaleneacetic acid, 98% purity) was obtained from West Asia Reagent Company. Acetonitrile (CR), methanol (CR), and ethanol (CR) were obtained from USA ACS Enke Chemical. FeSO_4_·7H_2_O (AR), NH_3_·H_2_O (AR), EDTA (AR), NaOH (AR), H_2_SO_4_ (AR), KI (AR), IPA (Isopropanol, AR), NaN_3_ (AR), and p-BQ (p-Benzoquinone, AR) were obtained from Shanghai Aladdin Bio-Chem Technology Co., Ltd. Ultrapure water employed in the experiments was obtained via an integrated Smart2 Pure ultrapure water system, obtained from TKA Wasseraufbereitungs system GmbH, Germany.

### Synthesis of Nanostructured γ-FeOOH

Freshly prepared under magnetic stirring, 10 ml of pure NH_3_·H_2_O was added to a 110 ml 0.3 mol·L^−1^ FeSO_4_ solution, which had a pH of 8.6. Subsequently, 10 ml of 0.015 mol·L^−1^ EDTA and ultrapure water were added to a 150-ml volume. Then, 1 L·min^−1^ O_2_ was introduced into the solution for about 30 min until the precipitation color changed from blue-green to orange under a controlled system temperature of 20°C, after which the pH was maintained at 4.3. Once the orange precipitate was filtered and rinsed, it was placed in a vacuum drying oven for 24 h at 30°C. Thereafter, the sample was finely ground and screened (200 mesh) (He et al., 2005).

### Characterization of Nanostructured γ-FeOOH

X-ray diffraction (XRD) was carried out with a Cu K(α) source (λ = 0.15406 nm) at 40 kV and 30 mA over the range of 2θ = 20–80°.

Scanning electron microscopy (SEM) was used for investigating the morphology and dispersion of the samples. Prior to measurements, the sample was affixed to an aluminum sheet and sprayed with gold.

BET surface area (BET) analysis was used to determine the pore structure, specific surface area, and porosity of the samples. The porosity and pore distribution were determined by a nitrogen adsorption–desorption isotherm and the Barrett–Joyner–Halenda (BJH) method.

### Photocatalytic NPX Degradation Experiments

#### Photoreaction Apparatus and Procedure

**Figure 1:**
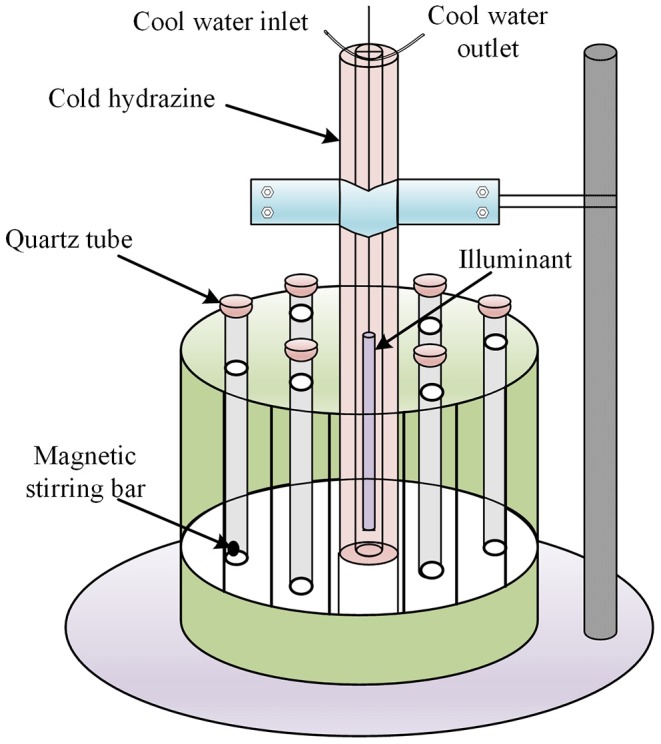
Multifunctional photochemical reaction instrument.

The photocatalytic NPX degradation experiments using γ-FeOOH were carried out using a multifunctional photochemical reaction instrument with magnetic stirring bars and a cooling circulation system (Figure 1). The illumination source in the experiment was a 300-W mercury lamp (Table 1), which was 10 cm away from the quartz tubes, and the temperature was held steady at 25°C during all tests. Prior to the photocatalytic degradation tests, the γ-FeOOH/NPX system was allowed to reach adsorption–desorption equilibrium in the dark for 240 min (He et al., 2005; Zhanyi et al., 2018). Subsequently, each experiment was conducted in triplicate with 20-ml samples under UV irradiation, and the rotary reactor was rotated at 5 rpm for 1 min, accompanied by constant magnetic stirring at 100 rpm for 1 min. A 10-ml sample was extracted via syringe every 2 min for each test and immediately passed through a 0.45-μm filter. The filtrate was then analyzed by high-performance liquid chromatography.

**Table 1:** Mercury lamp energy distribution.

**Wavelength** **λ (nm)**	**Relative energy** **(%)**	**Wavelength** **λ (nm)**	**Relative energy** **(%)**
1,367	15.3	289	6.0
1,129	12.6	280	9.3
1,014	40.6	275	2.7
577–579	76.5	270	4.0
546	93.0	265	15.3
436	77.5	257	6.0
405–408	42.2	254	16.6
365–366	100.0	248	8.6
334	9.3	240	7.3
313	49.9	238	8.6
302–303	23.9	236	6.0
297	16.6	232	8.0

#### Photodegradation of NPX by γ-FeOOH

To determine the effect of γ-FeOOH dosage, 0.05, 0.1, 0.2, 0.4, and 0.6 g·L^−1^ of γ-FeOOH was added to the NPX solution at a concentration of 10 mg·L^−1^.

To determine the effect of initial NPX concentration, NPX solutions with several concentrations of 5, 10, 20, 30, and 40 mg·L^−1^ were prepared, to which 0.2 g·L^−1^ of γ-FeOOH was added.

To determine the effect of pH, the pH was adjusted to 5, 7, and 9 by adding 0.1 mol·L^−1^ H_2_SO_4_ or 0.1 mol·L^−1^ NaOH to the NPX solution with a concentration of 10 mg·L^−1^, to which exactly 0.2 g·L^−1^ of γ-FeOOH was added.

The above experiments proceeded as described above.

#### Langmuir–Hinshelwood Kinetics

Heterogeneous photocatalysis includes two basic reaction steps, which are physical adsorption and a chemical reaction. For our experiments, Langmuir–Hinshelwood kinetics (L–H equation) were employed to fit the relationship between the photocatalytic reaction rate (*r*) and solution concentration (*C*):

(1) $$\begin{array}{l} r=\frac{dC}{dt}=k_{\text{L}-\text{H}}\frac{KC}{1+KC} \end{array}$$

where *r* is the initial photodegradation rate of NPX measured in mg·L^−1^·min^−1^, *k*_L−H_ is the photocatalytic degradation rate constant measured in mg·L^−1^·min^−1^, *K* is the adsorption constant of NPX on the surface of γ-FeOOH measured in L·mg^−1^, and *C* is the instantaneous concentration of NPX measured in mg·L^−1^.

There was a linear relationship between the reciprocal of *r* (1/*r*) and the reciprocal of *C* (1/*C*). Linear fitting was applied with the data from the experiment; hence, the photocatalytic degradation rate constant *k*_L−H_ and the adsorption constant *K* were obtained and found to be independent of the NPX concentration.

(2) $$\begin{array}{l} \frac{1}{r}\;=\;\frac{1}{k_{L-H}}\;+\;\frac{1}{k_{L-H}K}\cdot \frac{1}{C} \end{array}$$

#### Active Species Analysis

In order to investigate the role of free radicals in the photocatalytic degradation of NPX, radical quenching experiments were carried out. Four solutions were prepared, comprising 10 mg·L^−1^ NPX and 0.2 g·L^−1^ γ-FeOOH, to which 50 mmol·L^−1^ potassium iodide (KI), 0.10 mmol·L^−1^ isopropanol (IPA), 0.01 mmol·L^−1^ sodium azide (NaN_3_), and 0.01 mmol·L^−1^ benzoquinone (BQ) were added. In particular, KI was employed to quench the h^+^ and ·OH radicals (Zhang et al., 2011).

## Results and Discussion

### Characterization of γ-FeOOH

**Figure 2:**
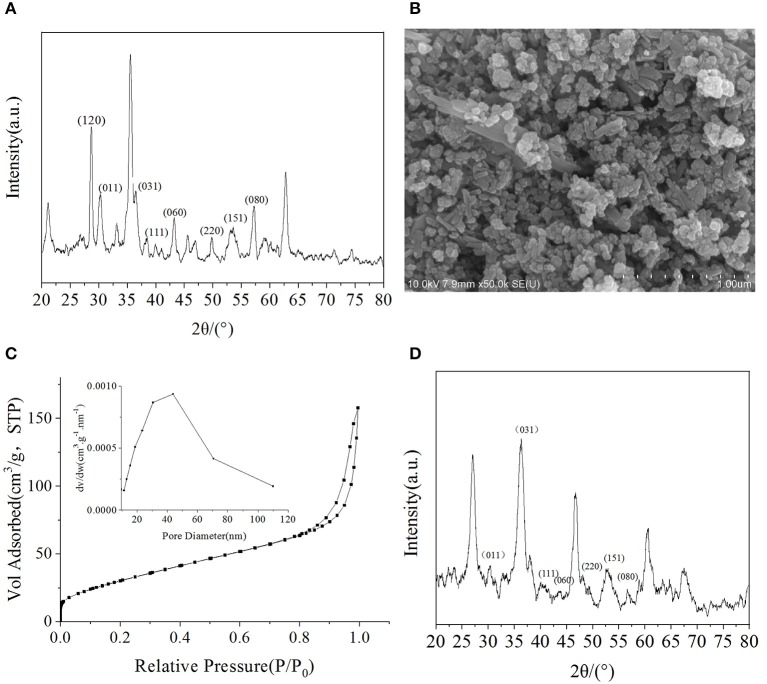
**(A)** XRD pattern of γ-FeOOH, **(B)** SEM image of γ-FeOOH, **(C)** N_2_ adsorption-desorption isotherm and pore size distribution of γ-FeOOH, **(D)** XRD pattern of recycled γ-FeOOH.

The physical properties of metal oxides, such as their crystal structures and surface characteristics, may influence their photocatalytic activity. Eight peaks were observed in the XRD pattern of FeOOH that were attributed to the (120), (011), (031), (111), (060), (220), (151), and (080) planes, indicating the presence of the γ structure (Figure 2A). When compared with the standard diffraction peaks of γ-FeOOH, the prepared powder was in the form of a pure crystal phase.

Surface morphological studies of γ-FeOOH by SEM revealed that the prepared powder was in the form of a mixed crystal phase, which contained, for the most part, nanoparticles (~50 nm) and short nanorods (~200 nm in length) (Figure 2B), due to differences in pH during the preparation of γ-FeOOH (Farcasiu et al., 1991). The smooth and dispersible properties of the mixed crystal phase revealed that the morphology was relatively regular.

The adsorption capacity of metal oxides for organic pollutants is affected by the BET size. On the basis of the N_2_ adsorption–desorption isotherm and pore size distribution, the specific surface area of γ-FeOOH was determined to be 125.7 m^2^·g^−1^ (Figure 2C). As shown in Figure 2C, we found that there was a significant hysteresis loop in the adsorption–desorption curve, which means that the sample possessed a mesoporous structure. According to the BJH desorption curve method (Kruk et al., 1997), which was employed to calculate the pore size distribution, the pore size range of the sample was ~50 nm. In photocatalytic experiments, γ-FeOOH was recovered and washed with pure water three times before drying. The XRD pattern showed no obvious change (Figure 2D) after this test. The degradation of NPX was reduced by only 1% when performing photodegradation with the recovered γ-FeOOH. These results show that the γ-FeOOH photocatalyst is highly stable.

**Figure 3:**
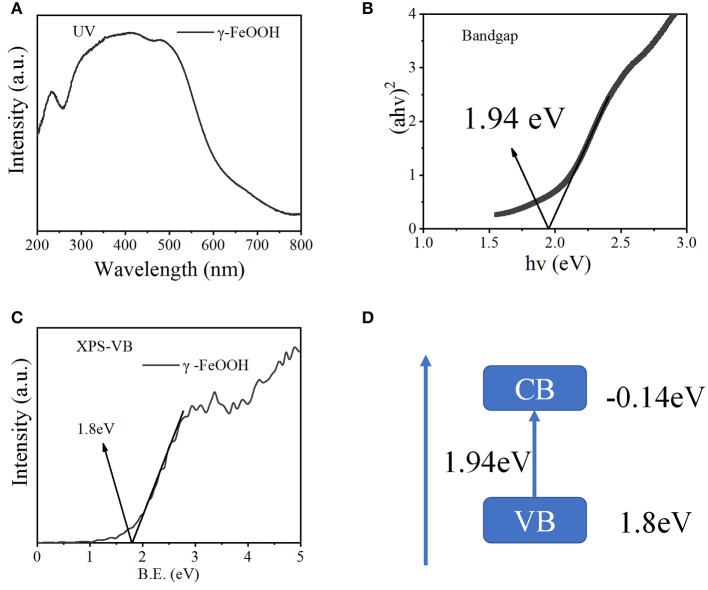
**(A)** UV-Vis diffuse spectra of γ-FeOOH, **(B)** bandgap width of γ-FeOOH, **(C)** XPS-VB spectra of γ-FeOOH, and **(D)** band structure alignments of γ-FeOOH.

As shown in Figure 3A, γ-FeOOH displayed a typical absorption edge at ~650 nm, and a bandgap width of 1.94 eV was calculated (Figure 3B). In order to further study the bandgap position of the semiconductor γ-FeOOH, X-ray photoelectron spectroscopy (XPS) was used to probe the valence band (XPS-VB). This revealed that the valence band of γ-FeOOH was located at 1.80 eV, as shown in Figure 3C. Therefore, it can be deduced that the rewind position of γ-FeOOH was −0.14 eV and the band gap structure is shown in Figure 3D.

### Effect of γ-FeOOH Dosage on the Photocatalytic Degradation of NPX

**Figure 4:**
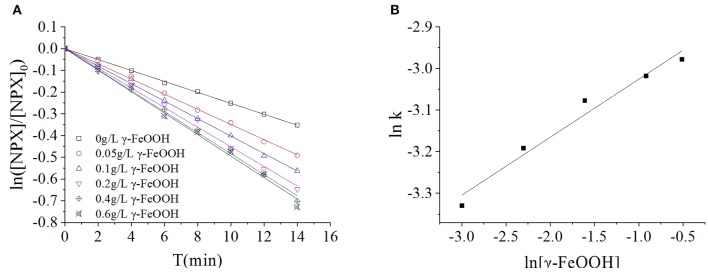
**(A)** Influence of γ-FeOOH dosage on NPX photodegradation, and **(B)** influence of γ-FeOOH dosage on the NPX photodegradation rate constant.

A suspension was formed in the multiphase photocatalytic reaction system, as the catalyst is insoluble in water. With increased catalyst dosages, the effective surface area of the solution was increased; hence, its reaction efficacy was enhanced proportionally. Excessive catalyst loading caused reflection and scattering, which reduced the transmittance of the solution and thus the catalytic efficiency. It was observed that the γ-FeOOH dosage played a very important role in the photodegradation of NPX. To investigate the effect of γ-FeOOH dosage on the photodegradation of NPX, γ-FeOOH solutions were prepared at concentrations of 0.05, 0.1, 0.2, 0.4, and 0.6 g·L^−1^, which were then introduced into separate NPX solutions. As shown in Figure 4A, the data collected from the photodegradation of NPX following the addition of different concentrations of γ-FeOOH were fitted to a first-order kinetic equation. It was observed that the NPX photodegradation rate increased with increased γ-FeOOH loading in water.

When the dosage of γ-FeOOH was varied from 0.05 to 0.6 g·L^−1^, the NPX photodegradation rate increased from 0.0344 to 0.0509 min^−1^. The position and photogenic charge of photocatalytic reactions in the system were enhanced with increased γ-FeOOH loading; however, the shielding, reflection, and scattering of light were increased with higher γ-FeOOH loads. With appropriate loads of γ-FeOOH, the transmittance of light in the solution decreased and the reaction rate slowly increased.

During the process of photodegradation, the relationship between the reaction rate constant *k* and the concentration of γ-FeOOH was fitted to the following empirical formula (Galindo et al., 2001):

(3) $$\begin{array}{l} k=a{\left[\gamma -FeOOH\right]}^{n} \end{array}$$

(4) $$\begin{array}{l} ln\;k=ln\;a+n\;ln\left[\gamma -FeOOH\right] \end{array}$$

where *n* is the correlation index and [γ-FeOOH] is the concentration of γ-FeOOH (g·L^−1^).

The kinetic constants of NPX photodegradation and the dosage of γ-FeOOH (0.05 to 0.6 g·L^−1^) in this experiment were analyzed by linear regression, with the relationship between *k* and [γ-FeOOH] shown in Figure 4B:

$$\begin{array}{l} ln\;k=ln\;0.05577+0.1394\;ln\left[\gamma -FeOOH\right]\;\left(R^{2}=0.9832\right) \end{array}$$

To: *k* = 0.05577[γ − *FeOOH*]^0.1394^

### Effect of Initial NPX Concentration on the γ-FeOOH/NPX System

It has been suggested that the charge transfer process between the contaminants adsorbed to the catalyst surface and the light-generated active species (h^+^, ·OH, and O_2_·) facilitates the photocatalytic oxidation of pollutants in solution. Therefore, the coverage of pollutants on the catalyst surface has an important influence on photocatalytic activity.

**Figure 5:**
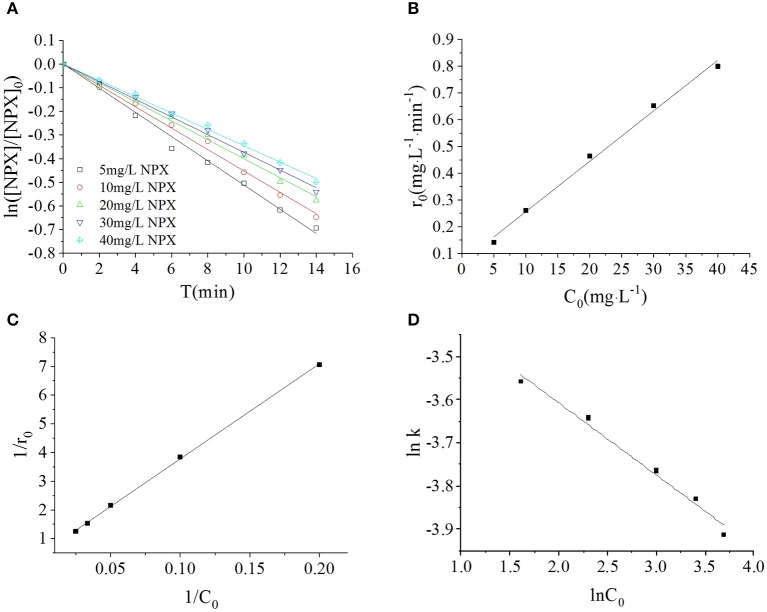
**(A)** Influence of initial NPX concentrations on the photodegradation of γ-FeOOH/NPX; **(B)** the initial reaction rate r_0_ as a function of initial NPX concentration C_0_; **(C)** Langmuir-Hinshelwood model of photocatalytic NPX degradation by γ-FeOOH; **(D)** influence of initial NPX concentrations on the photodegradation rate constant of γ-FeOOH/NPX.

In this section, the photodegradation of NPX by 0.2 g·L^−1^ γ-FeOOH was investigated at initial NPX concentrations of 5, 10, 20, 30, and 40 mg·L^−1^, with the results shown in Figure 5A. These experiments revealed that the photodegradation of NPX followed first-order kinetics at different initial concentrations upon the addition of γ-FeOOH. The activities of semiconductor photocatalysts arise primarily from photogenic e^−^ and h^+^, where, in the competitive process of photocatalysis, they may be recombined very rapidly (generally at the nanosecond level) (Hoffmann et al., 1995). From the kinetics perspective, only the adsorbents on the surface of the catalyst may be oxidized by e^−^. However, our results revealed that the NPX photodegradation rate decreased with higher initial concentrations in solution.

Under a certain light intensity, higher initial NPX concentrations resulted in a lower population of photons available per NPX molecule; hence, a lower photodegradation rate was obtained. An identical result was reported in previous NPX research (Ma et al., 2013). Secondly, higher initial NPX concentrations, with additional particles adsorbed to the γ-FeOOH surface, acted to lower the number of photocatalytically active sites that were available at the surface. Hence, the population of photogenerated e^−^/h^+^ pairs per unit of time was correspondingly reduced. Simultaneously, prior to the photodegradation of NPX molecules, they were required to undergo charge exchange with the active species generated at the γ-FeOOH surface and diffuse into the solution. Finally, when the initial NPX concentration was increased, it was difficult to completely decompose the reaction-generated intermediate products in a timely manner. This increased the opposition against adsorption to the surface of the γ-FeOOH, where these intermediates could once again reform the NPX matrix. Therefore, the photodegradation rate was finally decreased.

We considered the derivative of the obtained first-order kinetic equation with respect to *t* and set *t* = 0 to obtain the photodegradation rate *r*_0_ under different initial concentrations of *C*_0_, as shown in Figure 5B. When the initial concentration of NPX was increased from 5 to 40 mg·L^−1^, the initial photodegradation rate *r*_0_ also increased gradually, from 0.1415 to 0.7997 mg·L^−1^·min^−1^. This indicated that the photocatalytic degradation of NPX occurred on the surface of γ-FeOOH, and the photodegradation rate was an increasing function of the level of surface adsorption. When the Metastable-Equilibrium Adsorption Theory (Pan and Liss, 1998) is regarded under certain thermodynamic conditions, the adsorption amount is related to the surface binding strength and the adsorption configuration, while being balanced with the concentration of the solute. In this section, when the initial NPX concentration was raised, the coverage rate of the NPX molecules on the surface of γ-FeOOH increased accordingly. Consequently, the electron transfer efficiency of the NPX molecules that was adsorbed to the surface and the photogenerated charge were increased, which led to an increase of the initial photodegradation rate *r*_0_.

A large quantity of experimental data has indicated that the photocatalytic degradation of organic pollutants on the surface of semiconductors conforms to the Langmuir–Hinshelwood kinetic equation (Hoffmann et al., 1995; Houas et al., 2001; Andreozzi et al., 2003; Du et al., 2008; Li et al., 2008). The applicable premise of the L–H kinetic equation is that the organic pollutant molecules are adsorbed to a solid surface (Turchi and Ollis, 1990; Alfano et al., 1997). Although researchers have not clarified the photocatalytic mechanisms of FeOOH, surface complexes (Faust and Hoffmann, 1986) and semiconductor-initiated photocatalytic mechanisms (Bandana et al., 1999) have had their respective supporters. More recent studies have supported semiconductor photocatalytic mechanisms and highlighted the role of organic pollutant molecules adsorbed to the FeOOH surface (Bandana et al., 1999, 2001a,b). In examining the FeOOH-facilitated photocatalysis of orange II, Du et al. (2008) analyzed the initial reaction rate, amount of FeOOH surface adsorption, and the position of the FeOOH activity. Thus, Du considered that the FeOOH photocatalytic reaction takes place at the solid surface; therefore, the available L–H kinetic equation could be employed to describe FeOOH photocatalysis. Based on this, 1/*C*_0_ and 1/*r*_0_ were calculated according to Equation (2), and a plot was created for 1/*C*_0_-1/*r*_0_ (as shown in Figure 5C). A linear relationship was found between them within the experimental concentration range (*R*^2^ = 0.9996), *k*_L−H_ = 2.1867 mg·L^−1^·min^−1^, *K* = 0.01377 L·mg^−1^. This signified that the photocatalytic degradation of NPX on the surface of γ-FeOOH satisfies the L–H kinetic equation, and that the adsorption of NPX on γ-FeOOH is of importance to its photocatalytic degradation (Li et al., 2008).

Within the range of experimental concentrations, the photodegradation kinetic constant of NPX gradually decreased (from 0.0285 to 0.0200 min^−1^), whereas the correlation coefficient *R*^2^ decreased from 0.9949 to 0.9791. The relationship between the reaction rate constant *k* and the initial substrate concentration during the photocatalytic process could be generally described by the following empirical formula:

(5) $$\begin{array}{l} k=a{\left[NPX\right]}^{n} \end{array}$$

(6) $$\begin{array}{l} ln\;k=ln\;a+n\;ln\left[NPX\right] \end{array}$$

where *n* is the correlation index and [NPX] is the initial concentration of NPX (mg·L^−1^).

Linear regression was used to analyze the relationship between the NPX photodegradation kinetic constant and its initial concentration (5–40 mg·L^−1^) within the experimental range. It can be seen in Figure 5D that the relationship between the reaction rate constant *k* and NPX concentration was as follows:

$$\begin{array}{l} ln\;k=ln\;0.03790-0.1673\;ln\left[NPX\right]\;\left(R^{2}=0.9848\right)\\ \;k=0.03790{\left[NPX\right]}^{-0.1673} \end{array}$$

### Effect of pH on the γ-FeOOH/NPX System

It is understood that pH is a critical factor that influences the photocatalytic degradation kinetics during semiconductor multiphase photocatalysis. First, pH can change the charge properties of the catalyst surface and affect how organic molecules adsorb on the catalyst surface (Barnard et al., 2005). Secondly, photogenerated charge carriers can combine with H^+^/OH^−^ in solution to form active species, such as OH^−^, which can capture photogenerated holes H^+^ to form ·OH. Finally, pH may alter the electron cloud density distribution of organic molecules, thus affecting photocatalytic degradation.

**Figure 6:**
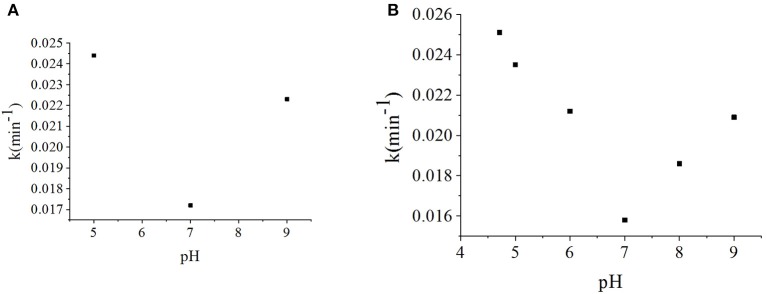
**(A)** Influence of the pH value on photodegradation rate constant of γ-FeOOH/NPX, and **(B)** influence of the pH value on photodegradation of NPX.

As the effects of pH on photocatalytic degradation kinetics are relatively complex, definitive studies of γ-FeOOH are relatively rare. In this section, to investigate the effects of initial pH on the photodegradation of γ-FeOOH/NPX reaction systems, the initial NPX concentration was established as 10 mg·L^−1^, whereas that of γ-FeOOH was 0.2 g·L^−1^, and the initial pH of the photodegradation solution was set at 5, 7, and 9. As shown in Figure 6A, at a pH of 5, the γ-FeOOH /NPX system demonstrated the fastest photocatalytic rate with a pH of 9 in the second place, while the slowest rate was observed at a pH of 7, much the same as the photocatalytic rates observed in water (Figure 6B).

As pH_ZPC_ = 8.47 for γ-FeOOH, the hydroxylation of the γ-FeOOH surface in an alkaline solution could allow OH^−^ to react with h^+^ to produce ·OH as follows:

(7) $$\begin{array}{l} \text{F}{\text{e}}^{\text{III}}-\text{O}{\text{H}}^{-}+{\text{h}}^{+}\to \text{F}{\text{e}}^{\text{III}}+\cdot \text{OH} \end{array}$$

Concurrently, the ether bonds of the NPX molecules are less stable under acidic conditions (Chen et al., 2013). Hence, γ-FeOOH was favorable for the photocatalytic degradation of NPX at pH < 7.

Based on the above analysis, when the pH was low, the stability of the ether bonds within the NPX molecules was decreased, which enabled the γ-FeOOH-based photocatalytic degradation of NPX. When the pH was high, OH^−^ combined with h^+^ to form ·OH, which facilitated the photocatalytic degradation of NPX. Due to the combined effect of these two factors, the reaction rate was lowest when the pH was 7 within the range of our experiments.

### Analysis of the Photocatalytic Degradation Mechanism of γ-FeOOH

**Figure 7:**
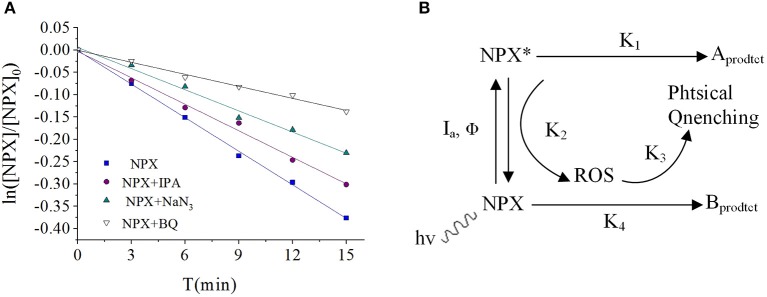
**(A)** Influence of scavengers on the NPX photodegradation rate constant, and **(B)** photodegradation of NPX in the water.

Quenching experiments were carried out (Figure 7A) by measuring the generation of active species during the photodegradation of NPX in pure water. It can be seen that there was not only direct photodegradation caused by ^3^NPX^*^, but also self-sensitized photodegradation involving hydroxyl radicals (·OH), singlet oxygen (^1^O_2_), and superoxide anions (${\text{O}}_{2}^{\cdot -}$) which were produced in the photodegradation process of NPX (Figure 7B) (Zhanyi et al., 2018).

**Figure 8:**
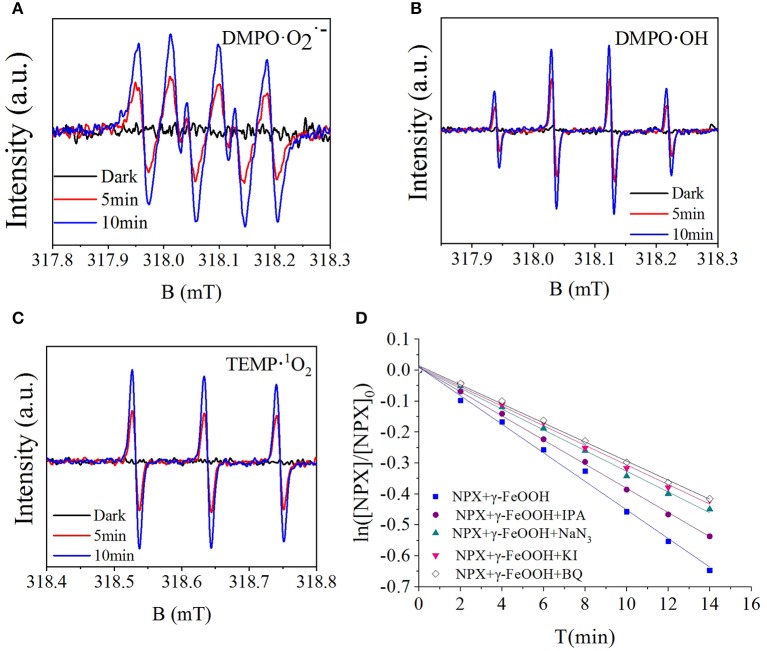
**(A)** ESR spectra of the DMPO-${\text{O}}_{2}^{\cdot -}$, **(B)** DMPO-·OH, **(C)** TEMP-^1^O_2_, and **(D)** influence of scavengers on the γ-FeOOH/NPX photodegradation rate constant.

To further investigate the active radicals that participate in the photodegradation of NPX, electron paramagnetic resonance (EPR) measurements were carried out. As shown in Figure 8A, there was no signal in the dark, while the signal 1:1:1:1 appeared after 5 min of illumination. It could thus be concluded that ${\text{O}}_{2}^{\cdot -}$ was present and its concentration would be increased by illumination. As shown in Figure 8B, it was observed that the signal 1:2:2:1 appeared. This suggests that ·OH appeared and increased in concentration with illumination. It was also confirmed that ^1^O_2_ was present by TEMP from Figure 8C, while the signal 1:1:1 was detected in the light. So, the active radicals in the γ-FeOOH/NPX system were evidenced by electron spin resonance (ESR).

Photocatalytic degradation typically generates a variety of active substances, such as h^+^, e^−^, ·OH, ^1^O_2_, and ${\text{O}}_{2}^{\cdot -}$ (Hao et al., 2016), with the production processes shown in Equations (8)–(14):

(8) $$\begin{array}{l} \text{γ}-\text{ FeOOH}+\text{hv}\to {\text{h}}^{+}+{\text{e}}^{-} \end{array}$$

(9) $$\begin{array}{l} {\text{h}}^{+}+{\text{H}}_{2}\text{O}\to \cdot \text{OH}+{\text{H}}^{+} \end{array}$$

(10) $$\begin{array}{l} {\text{e}}^{-}+{\text{O}}_{2}\to {\text{O}}_{2}^{\cdot -} \end{array}$$

(11) $${\text{O}}_{2}^{\cdot -}+{\text{H}}^{+}+\to {\text{HO}}_{2}^{\cdot }$$

(12) 2HO2·→H2O2+O21

(13) $$\begin{array}{l} {\text{H}}_{2}{\text{O}}_{2}+{\text{e}}^{-}\to \cdot \text{OH}+\text{O}{\text{H}}^{-} \end{array}$$

(14) $$\begin{array}{l} \text{Active substances}+\text{NPX}\to \text{Products} \end{array}$$

According to the quenching experiment described in the section “Active Species Analysis,” when KI, IPA, NaN_3_, and BQ were added to the solution, the photocatalytic NPX degradation rate was reduced by different degrees, as shown in Figure 8D. It may be seen from Figure 8D that free radicals such as h^+^, e^−^, ·OH, ^1^O_2_, and ${\text{O}}_{2}^{\cdot -}$ were involved in the γ-FeOOH-mediated photocatalytic degradation of NPX.

On one hand, γ-FeOOH is a semiconductor material with an energy band structure, where the energy barrier (Eg) between the valence band (VB) and the conduction band (CB) is only 2.2 eV. When the γ-FeOOH surface was irradiated with photons of energy equal to or greater than the forbidden band, the e^−^ in the VB were excited and jumped to the CB, with h^+^ being generated in the VB to form e^−^/h^+^ pairs. Because of the discontinuous region between the energy bands, the resulting e^−^/h^+^ pairs had greater longevity; hence, they migrated to the particle surface in large quantities. The oxidizing properties of the nanoparticle surfaces were potent enough to oxidize the NPX molecules that were adsorbed to the γ-FeOOH surfaces. Additionally, the cavities reacted with H_2_O molecules, which were also attached to the surface of γ-FeOOH, which then generated ·OH. Due to the strong oxidization ability of ·OH, the NPX molecules on the surface of γ-FeOOH could also be oxidized and degraded. Simultaneously, conducting electrons were combined with O_2_ at the surface of the γ-FeOOH to generate ${\text{O}}_{2}^{\cdot -}$, which could also facilitate the oxidative degradation of NPX.

On the other hand, under sunlight exposure, Fe (III) could accelerate the oxidation of carboxylic acid. As NPX contains a carboxyl group, it could form strong complexes with Fe (III), which rapidly photochemically reacted under light irradiation (Zuo and Hoigne, 1992; Faust and Zepp, 1993), thereby accelerating the oxidative degradation of NPX. Several studies have suggested that the photochemical reaction of these complexes follows the H_2_O_2_ production process in water.

(15) $$\text{Fe}\left({\text{OX}}_{\text{n}}^{\left(3-2\text{n}\right)+}\right)+\text{hv}\to \text{Fe}\left({\text{OX}}_{\text{n}-\text{1}}^{\left(\text{4}-\text{2n}\right)+}\right)+{\text{OX}}^{\cdot -}$$

(16) $$\begin{array}{l} \text{O}{\text{X}}^{\cdot -}+{\text{O}}_{2}\to {\text{O}}_{2}^{\cdot -}+2\text{C}{\text{O}}_{2} \end{array}$$

(17) $${\text{O}}_{2}^{\cdot -}+{\text{H}}^{+}\to {\text{HO}}_{2}^{\cdot }$$

(18) $${\text{HO}}_{2}^{\cdot }/{\text{O}}_{2}^{\cdot -}+\text{Fe}\left(\text{III}\right)+{\text{H}}^{+}\to \text{Fe}\left(\text{II}\right)+{\text{O}}_{2}$$

(19) $${\text{HO}}_{2}^{\cdot }/{\text{O}}_{2}^{\cdot -}+\text{Fe}\left(\text{II}\right)+{\text{H}}^{+}\to \text{Fe}\left(\text{III}\right)+{\text{H}}_{2}{\text{O}}_{2}$$

For this photodegradation experiment, the effect of hydrokinetics must also be considered, as light exposure under stirring was first applied. With agitation, the mass transfer rate of NPX from the solution to the γ-FeOOH surface was increased, so additional NPX was oxidized prior to e^−^/h^+^ recombination and thus the photodegradation of NPX was increased. Moreover, DO present in the solution could capture the photogenerated electrons generated during the photocatalytic process, which reduced the probability of the recombination of photogenerated electrons and holes, and thus increased the probability of holes oxidizing the NPX. When exposed to UV light, the e^−^ at the surface of the γ-FeOOH could reduce O_2_ to ${\text{O}}_{2}^{\cdot -}$, as shown in Equation (20). Subsequently, ${\text{O}}_{2}^{\cdot -}$ reacted with photogenerated holes h^+^ to form ·OH or peroxide in the presence of organic capture agents, as in Equations (21)–(23). Each of these species contributed to the photodegradation of NPX.

(20) $${\text{e}}^{-}+{\text{O}}_{2}\to {\text{O}}_{2}^{\cdot -}$$

(21) $$2{\text{O}}_{2}^{\cdot -}+2{\text{H}}^{+}\to {\text{O}}_{2}+{\text{H}}_{2}{\text{O}}_{2}$$

(22) $$\begin{array}{ll} {\text{H}}_{2}{\text{O}}_{2}+{\text{e}}^{-} & \to \text{HO}\cdot +\text{O}{\text{H}}^{-} \end{array}$$

(23) $${\text{O}}_{2}^{\cdot -}+\text{NPX }\to \text{NPX}-{\text{OO}}^{\cdot }$$

### Identification of Intermediates

**Figure 9:**
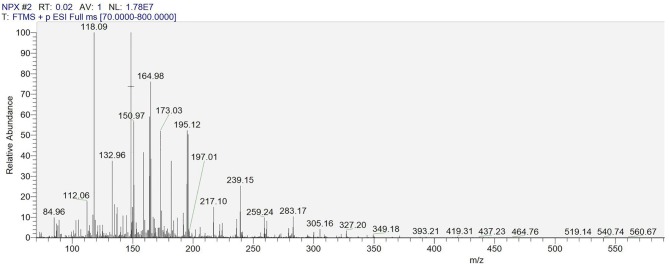
HRLC-MS-MS spectrum of the intermediates on the γ-FeOOH/NPX system.

**Figure 10:**
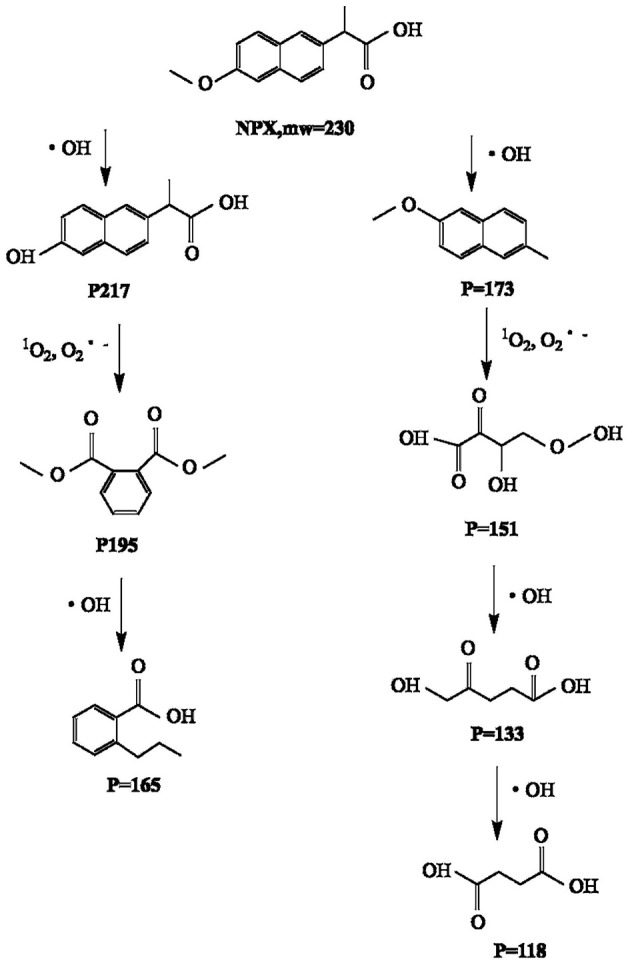
Speculated degradation approach of NPX on the γ-FeOOH/NPX system.

The degradation by-products of NPX on the γ-FeOOH /NPX system were identified by Thermo Scientific Ultimate 3000 RSLC and Q Exactive Orbitrap (HRLC-MS-MS). As shown in Figure 9, seven intermediates were detected. From attacked by h^+^, e^−^, ·OH, ^1^O_2_, and ${\text{O}}_{2}^{\cdot -}$, compounds were generated because of the losses of the CO_2_, H_2_O, and/or CH_3_ group. According to the deduced structure of the compounds and the early study, we speculated the reasonable reaction approach as shown in Figure 10.

## Conclusions

This study concludes that the photodegradation rate of NPX was positively correlated with the concentration of γ-FeOOH in solution, which was related to the absorption of light energy. With increased initial concentrations of NPX, the photodegradation rate decreased while the γ-FeOOH concentration was constant. This was because the population of photons available per NPX molecule was reduced due to the invariable intensity of light, and the numbers of e^−^/h+ pairs generated on the surface of the γ-FeOOH were reduced per unit. At the same time, the intermediate products generated by the reaction could not be completely decomposed in time, so they engaged in a reverse reaction to reconstitute the NPX matrix. At the tested pH values (5.0, 7.0, and 9.0), the photocatalytic rate was noticeably accelerated at higher and lower pH, while the worst pH for photocatalysis was 7.0. Based on quenching experiments and analysis of the photocatalytic mechanism, we conclude that the photocatalysis of NPX degradation by γ-FeOOH is derived from semiconductor photocatalysis. At last, the intermediates of NPX on the γ-FeOOH /NPX System were identified by HRLC-MS-MS.

## Data Availability Statement

All datasets generated for this study are included in the manuscript.

## Author Contributions

ZL and GL formulated the problem and designed the experiments. ZL, XJ, and XW performed the experiments. ZL and QS took part in data collection and analysis and wrote the paper. QS and CL revised the manuscript.

### Conflict of Interest

The authors declare that the research was conducted in the absence of any commercial or financial relationships that could be construed as a potential conflict of interest.

## References

[B1] Alfano O. M. Cabrera M. I. Cassano A. E. (1997). Photocatalytic reactions involving hydroxyl radical attack. J. Catal. 172, 370–379. 10.1006/jcat.1997.1858

[B2] Andreozzi R. Caprio V. Marotta R. (2003). Iron (III)(hydr) oxide-mediated photooxidation of 2-aminophenol in aqueous solution: a kinetic study. Water Res. 37, 3682–3688. 10.1016/S0043-1354(03)00271-9 12867335

[B3] Bandana J. Mielczarski J. Kiwi J. (1999). Molecular mechanism of surface recognition. azo degradation on Fe, Ti, and Al oxides through metal sulfonate complexes. Langmuir 15, 767–7679. 10.1021/la9900270

[B4] Bandana J. Mielczarski J. A. Lopez A. Kiwi J. (2001a). Sensitized degradation of chlorophenols on iron oxides induced by visible light : Comparison with titanium oxide. Appl. Catal. B Environ. 34, 321–333. 10.1016/S0926-3373(01)00225-9

[B5] Bandana J. Tennakone K. Kiwi J. (2001b). Surface mechanism of molecular recognition between aminophenols and iron oxide surfaces. Langmuir 17, 3964–3969. 10.1021/la001411w

[B6] Barnard A. S. Zapol P. Curtiss L. (2005). Anatase and ruble surface with adsobates representative of acidic and basic conditions. Surf. Sci. 582, 173–188. 10.1016/j.susc.2005.03.014

[B7] Chen Y. L. Liu G. G. Yao K. Lü W. (2013). Treatment of naproxen-containing water in low concentration by ultraviolet irradiation. Chin. J. Environ. Eng. 2, 473–476. Available online at: http://www.cjee.ac.cn/article/id/20130212

[B8] Christina I. K. Dimitra A. L. Triantafyllos A. A. (2014). Investigation of PPCPs in wastewater treatment plants in Greece: Occurrence, removal and environmental risk assessment. Sci. Total Environ. 466–467, 421–438. 10.1016/j.scitotenv.2013.07.044 23933429

[B9] Dai G. Huang J. Chen W. Wang B. Yu G. Deng S. (2014). Major pharmaceuticals and personal care products (PPCPs) in wastewater treatment plant and receiving water in Beijing, China, and associated ecological risks. B Environ. Contam. Tox. 92, 655–661. 10.1007/s00128-014-1247-0 24619361

[B10] Domínguez J. R. González T. Palo P. Cuerda-Correa E. M. (2011). Removal of common pharmaceuticals present in surface waters by Amberlite XAD-7 acrylic-ester-resin: influence of pH and presence of other drugs. Desalination 269, 231–238. 10.1016/j.desal.2010.10.065

[B11] Du W. Xu Y. Wang Y. (2008). Photoinduced degradation of orange II on different iron (hydr) oxides in aqueous suspension: rate enhancement on addition of hydrogen peroxide, silver nitrate, and sodium fluoride. Langmuir 24, 175–181. 10.1021/la7021165 18052220

[B12] Farcasiu M. Smith C. Pradhan V. R. Wender I. (1991). Iron compounds and iron catalysts: activity in reactions relevant to direct coal liquefaction. Fuel Process. Technol. 29, 199–208. 10.1016/0378-3820(91)90036-C

[B13] Faust B. C. Hoffmann M. R. (1986). Photoinduced reductive dissolution of a-Fe_2_O_3_ by bisulfate. Environ. Sci. Technol. 20, 943–948. 22263829 10.1021/es00151a015

[B14] Faust B. C. Zepp R. G. (1993). Photochemistry of aqueous iron (III)-polycarboxylate complexes: roles in the chemistry of atmospheric and surface waters. Environ. Sci. Technol. 27, 2517–2522. 10.1021/es00048a032

[B15] Fortin D. Langley S. (2005). Formation and occurrence of biogenic iron- rich minerals. Earth Sci. Rev. 72. 1–19. 10.1016/j.earscirev.2005.03.002

[B16] Galindo C. Jacques P. Kalt A. (2001). Photooxidation of the phenylazonaphthol AO20 on Ti0_2_: kinetic and mechanist investigations. Chemosphere 45, 997–1005. 10.1016/S0045-6535(01)00118-7 11695623

[B17] Hao R. Wang G. Tang H. (2016). Template-free preparation of macro/mesoporous g-C_3_N_4_/TiO_2_ heterojunction photocatalysts with enhanced visible light photocatalytic activity. Appl. Catal. B Environ. 187, 47–58. 10.1016/j.apcatb.2016.01.026

[B18] Hasan Z. Jeon J. Jhung S. H. (2012). Adsorptive removal of naproxen and clofibric acid from water using metal-organic frameworks. J. Hazard. Mater. 209–210, 151–157. 10.1016/j.jhazmat.2012.01.005 22277335

[B19] He Y. P. Miao Y. M. Li C. R. Wang Q. Cao L. Xie S. S. (2005). Size and structure effect on optical transitions of iron oxide nanocrystals. Phys. Rev. B. 71:125411 10.1103/physrevb.71.125411

[B20] Hoffmann M. R. Martin S. T. Choi W. Bahnemann D. W. (1995). Environmental applications of semiconductor photocatalysis. Chem. Rev. 95, 69–96. 10.1021/cr00033a004

[B21] Houas A. Lachheb H. Ksibi M. Elaloui E. Guillard C. Herrmann J. M. (2001). Photocatalytic degradation pathway of methylene blue in water. Appl. Catal. B Environ. 31, 145–157. 10.1016/S0926-3373(00)00276-9

[B22] Isidori M. Lavorgna M. Nardelli A. Parrella A. Previtera L. Rubino M. (2005). Ecotoxicity of naproxen and its phototrans formation products. Sci. Total Environ. 348, 93–101. 10.1016/j.scitotenv.2004.12.068 16162316

[B23] Jones O. A. H. Voulvorlis N. Lester J. N. (2002). Aquatic environmental assessment of the top 25 English prescription pharmaceuticals. Water Res. 36, 5013–5022. 10.1016/S0043-1354(02)00227-0 12448549

[B24] Karl F. Anna A. W. Daniel D. (2006). Ecotoxicology of human pharmaceuticals. Aquat. Toxicol. 76, 122–159. 10.1016/j.aquatox.2005.09.009 16257063

[B25] Kruk M. Jaroniec M. Sayari A. (1997). Application of large pore MCM-41 molecular sieves to improve pore size analysis using nitrogen adsorption measurements. Langmuir 13, 6267–6273. 10.1021/la970776m

[B26] Lee A. P. Brooker L. R. Macey D. J. van Bronswijk W. Webb J. (2000). Apatite mineralization in teeth of the chiton acanthopleura echinata. Calc. Tissue Int. 67, 408–415. 10.1007/s002230001156 11136540

[B27] Li Y. Sun S. Ma M. Ouyang Y. Yan W. (2008). Kinetic study and model of the photocatalytic degradation of rhodamine B (RhB) by a TiO_2_-coated activated carbon catalyst: effects of initial RhB content, light intensity and TiO_2_ content in the catalyst. Chem. Eng. J. 142, 147–155. 10.1016/j.cej.2008.01.009

[B28] Ma D. J. Liu G. G. Lü W. Y. Yao K. Zhou L. H. Xie C. P. (2013). Photodegradation of Naproxen in aqueous systems by UV irradiation: mechanism and toxicity of photolysis products. Environ. Sci. 34, 1782–1789. Available online at: http://www.hjkx.ac.cn/hjkx/ch/reader/view_abstract.aspx?flag=1&file_no=20130517&journal_id=hjkx 23914528

[B29] Méndez-Arriaga F. Gimenez J. Esplugas S. (2008). Photolysis and TiO_2_ photocatalytic treatment of naproxen: degradation, mineralization, intermediates and toxicity. J. Adv. Oxid. Technol. 3, 435–444. 10.1515/jaots-2008-0302

[B30] Molgaard J. (1974). Corrosion of cast iron in chlorinated sea water, in 5th International Congress on Metallic Corrosion (Tokyo), 792–795.

[B31] Nurmi J. T. Tratnyek P. G. Sarathy V. Baer D. R. Amonette J. E. Pecher K.. (2005). Characterization and properties of metallic iron nanoparticles: Spectroscopy, electrochemistry, and kinetics. Environ. Sci. Technol. 39, 1221–1230. 10.1021/es049190u 15787360

[B32] Pan G. Liss P. S. (1998). Metastable-equilibrium adsorption theory: II. Experimental. J. Coll. Interface Sci. 201, 71–76. 10.1006/jcis.1998.5397

[B33] Turchi C. S. Ollis D. F. (1990). Photocatalytic degradation of organic water contaminants: Mechanisms involving hydroxyl radical attack. J. Catal. 122, 178–192. 10.1016/0021-9517(90)90269-P

[B34] Xu J. Wu L. Chang A. C. (2009). Degradation and adsorption of selected pharmaceuticals and personal care products (PPCPs) in agricultural soils. Chemosphere 77, 1299–1305. 10.1016/j.chemosphere.2009.09.063 19853275

[B35] Zhang N. Liu G. Liu H. Wang Y. He Z. Wang G. (2011). Diclofenac photodegradation under simulated sunlight: Effect of different forms of nitrogen and Kinetics. J. Hazard. Mater. 192, 411–418. 10.1016/j.jhazmat.2011.05.038 21664047

[B36] Zhanyi L. Guoguang L. Qing S. Jin X. Wen X. Zhang G. (2018). Kinetics and thermodynamics of NPX adsorption by γ-FeOOH in aqueous media. Arab. J. Chem. 11, 910–917. 10.1016/j.arabjc.2018.02.005

[B37] Zheng B. G. Niu J. L. Zheng Z. Zhang J. Luo X. Zhao Y. (2011). Catalytic degradation of Naproxen in aqueous solutions by gamma-ray irradiation. Environ. Chem. 12, 2022–2025. 10.1007/s11783-010-0264-4

[B38] Zhou S. G. Zhou L. X. Chen F. X. (2007). Characterization and heavy metal adsorption properties of Schwertmannite synthesized by bacterial oxidation of ferrous sulfate solutions. Spectr. Spectr. Anal. 27, 367–370. 10.1016/j.sab.2007.02.003 17514978

[B39] Zuo Y. Hoigne J. (1992). Formation of hydrogen peroxide and depletion of oxalic acid in atmospheric water by photolysis of iron (III)-oxalato complexes. Environ. Sci. Technol. 26, 1014–1022. 10.1021/es00029a022

